# Supplementary material for: Mapping the cardiac vascular niche in heart failure
Source: Nat Commun. 2022 May 31;13:3027. doi: 10.1038/s41467-022-30682-0 (PMC9156759; doi:10.1038/s41467-022-30682-0)
Supplement: Supplementary file 1 — Supplementary Information [file 41467_2022_30682_MOESM1_ESM.pdf]

Supplementary Information

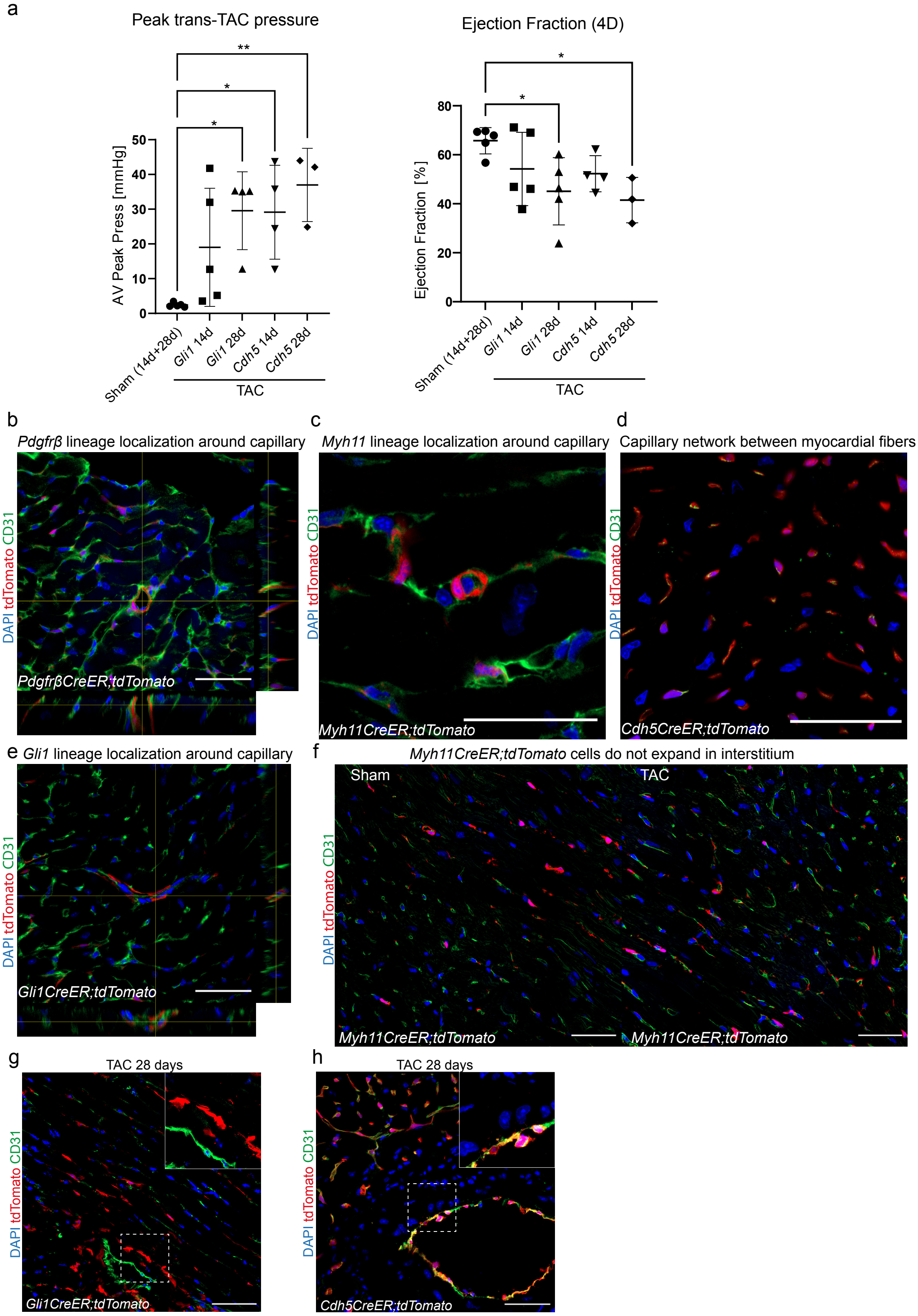

Supplementary Fig. 1: Functional evaluation of TAC model using echocardiography and confocal images visualizing cardiac localization of fate traced cells

a, Echocardiography measurement of cardiac function acquired by pulse wave doppler and four dimensional (4D) whole heart imaging. Left panel, peak trans-TAC pressure calculated from Doppler velocities using the Bernoulli equation[1]. Right panel, ejection fraction calculated from 4D volume measurement. Sham group combined for genotype and time point (mean ± SD; n = 3 sham mice, n = 5 TAC Gli1 mice, n = 5 TAC Cdh5 mice; all mice

examined over two independent time points. *: p-value < 0.05, **: p-value < 0.01, one-way ANOVA with Dunnett's post-hoc). Source data are provided as a Source Data file. **b**, Representative orthogonal view of tdTomato^{+} cells from *PdgfrβCreER;tdTomato* heart (20 μm z-stack). **c**, Representative image of interstitial perivascular tdTomato^{+} fate traced cells from *Myh11CreER*. **d**, Representative image of *Cdh5CreER* fate traced cells in the cardiac interstitium. **e**, Representative orthogonal view of *Gli1CreER;tdTomato* heart (20 um z-stack). **f,** Representative images comparing interstitial localization of *Myh11CreER* fate traced cells between sham and TAC 14 day hearts. All scale bars in this figure represent 50 μm. **g-h,** Representative stainings visualizing localization of fate traced cell types (tdTomato, red) after 28 days TAC (scale bars 50 μm).

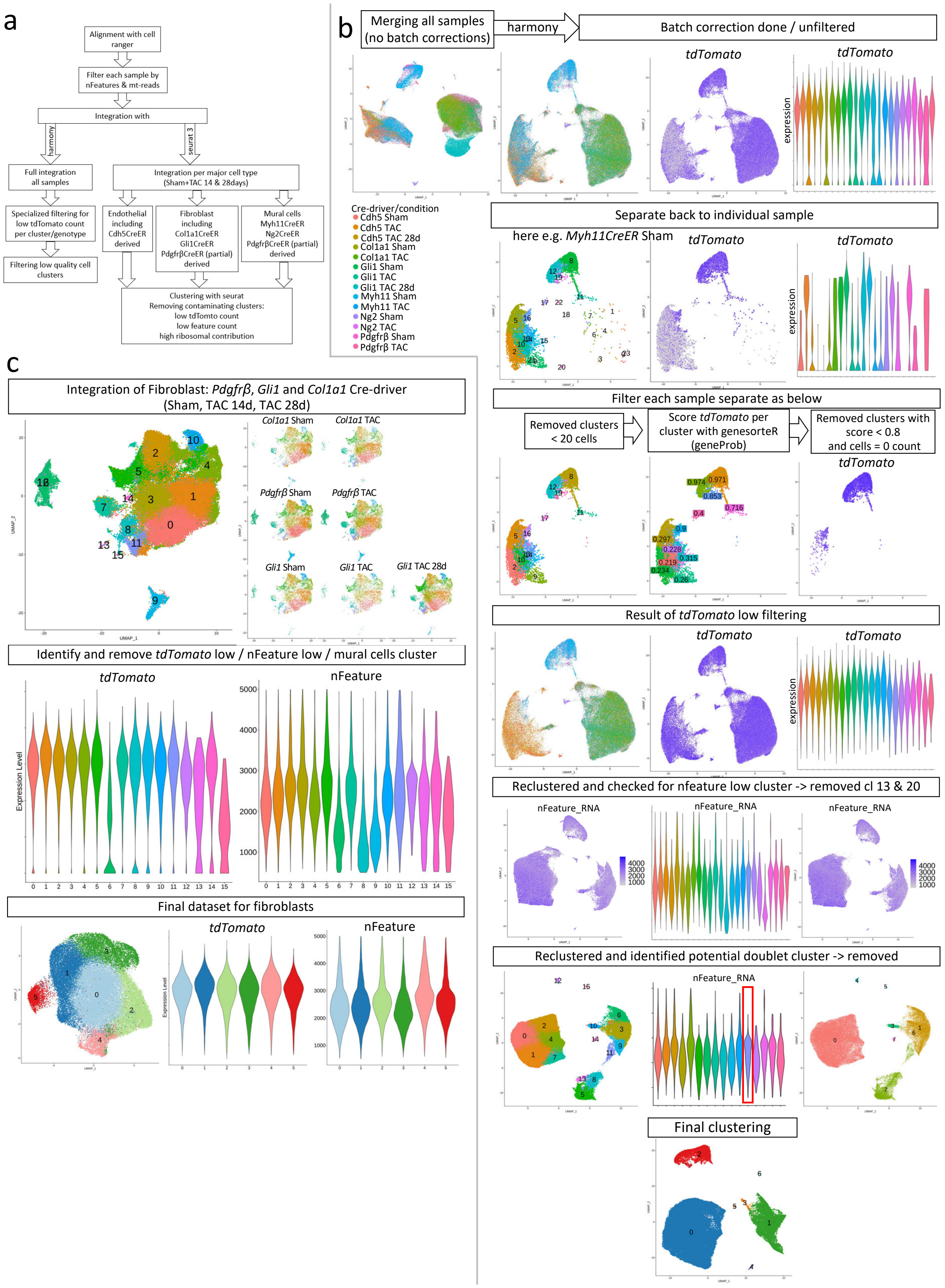

Supplementary Fig. 2: Strategy for analyzing the single cell RNA sequencing data and processing of the full dataset integration and fibroblast subtype integration

a, Schematic overview of the general steps in the data processing, which was applied to all samples. Raw sequencing files were aligned to the mouse genome (including *tdTomato* gene) using cell ranger count function. Each dataset was filtered as indicated before integration. Seurat canonical correlation analysis was used for integration of samples by major cell type (fibroblast, EC, mural cells). Harmony based batch correction was used

for integrating all datasets. **b**, Stepwise representation of data processing and filtering of the full integration with all 14 datasets generated in the study. Batch correction with harmony was applied as the first step to correct for technical batch effects of the individual samples. The dataset was clustered based on the dimensional reduction from harmony and filtered using the gene expression of the lineage tracing gene *tdTomato*. Therefore, the dataset was split back to the individual sample, while keeping the UMAP embedding structure and clustering. Clusters with less than 20 cells were then removed per sample, as well as clusters with a conditional gene probability for *tdTomato* (calculated by genesorterR function sortGenes) below 0.8 per sample. Remaining cells with 0 reads for *tdTomato* were removed afterwards and the full dataset was reclustered based on the *tdTomato* filtered dataset. Clusters of low feature count were identified as low-quality cell clusters and removed, same as clusters which were identified with a high likelihood as contaminating doublets, based on overlap of marker gene expression and unusual high feature count. **c**, Stepwise representation of data processing and filtering of the integration of all samples contributing to the fibroblast analysis. All samples containing fibroblasts (*Col1a1CreER*, *Gli1CreER*, *PdgfrβCreER*) were integrated using canonical correlation analysis from the Seurat package and filtered for clusters with low *tdTomato* expression and low feature count. From the *PdgfrβCreER* datasets only cells identified as fibroblasts were included for the integration.

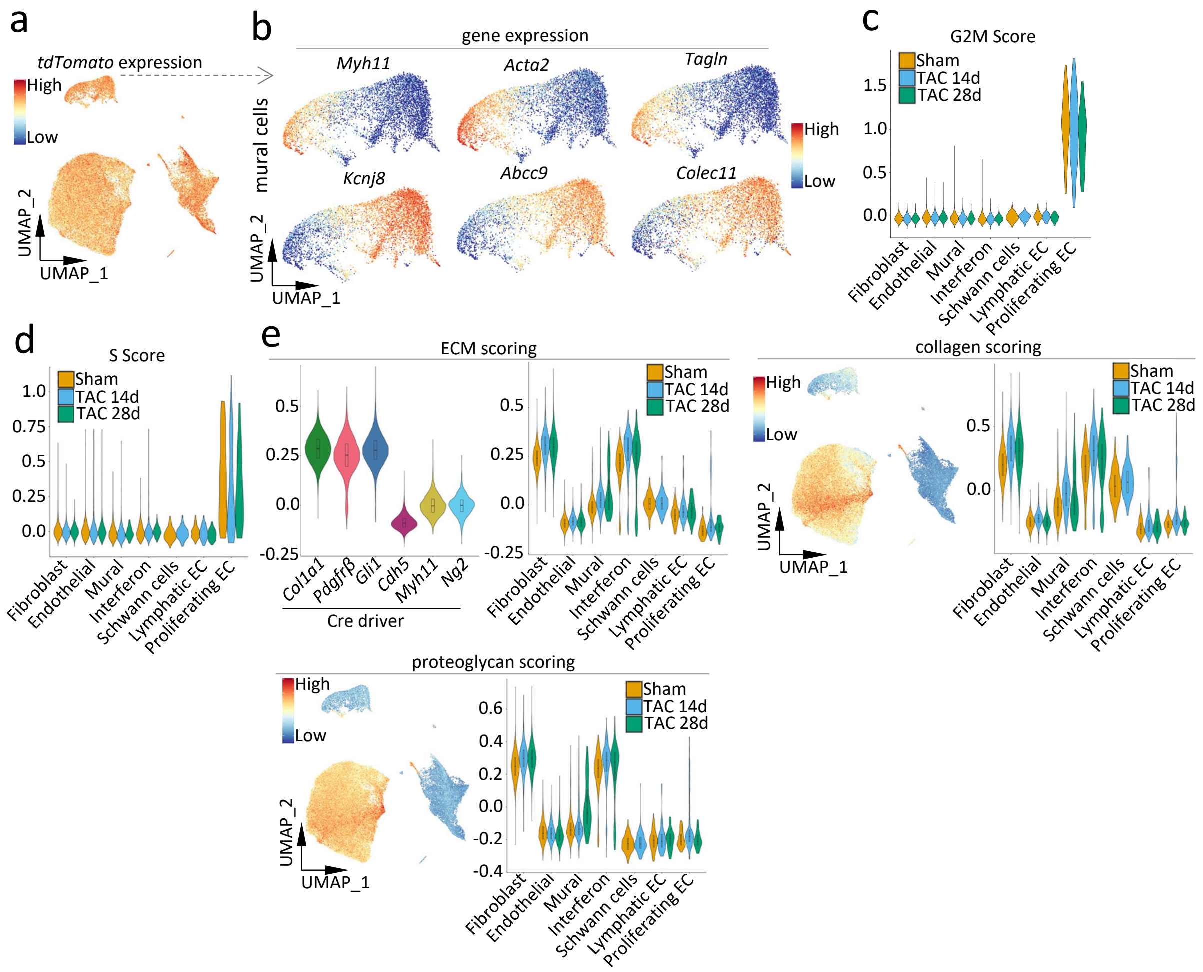

Supplementary Fig. 3: Comprehensive analysis of full integration dataset

a, UMAP embedding of the filtered full integration showing the *tdTomato* gene expression per cell. Color indicates level of expression. **b,** UMAP embedding of the mural cell cluster from the full integration, indicated by arrow. Gene expression of indicated genes is displayed per cell. **c-d**, Violin plot of summarized G2M and S scores per condition and cell type, based on the Seurat cell cycle scoring vignette. **e**, Extracellular matrix (ECM), collagen and proteoglycan gene set scoring. For ECM score: Left, violin plot of scores summarized per lineage tracing genotype; right, summarized per cluster and condition. For collagen and proteoglycan score: Left, UMAP embedding showing collagen score per cell; right, violin plot of scores summarized per cluster and condition. Integrated boxplots show center line as median, box limits as upper and lower quartiles. Groups in violin plots were tested for significant differences between conditions (two-sided Wilcoxon rank sum test, unpaired, Bonferroni adjusted p-value).

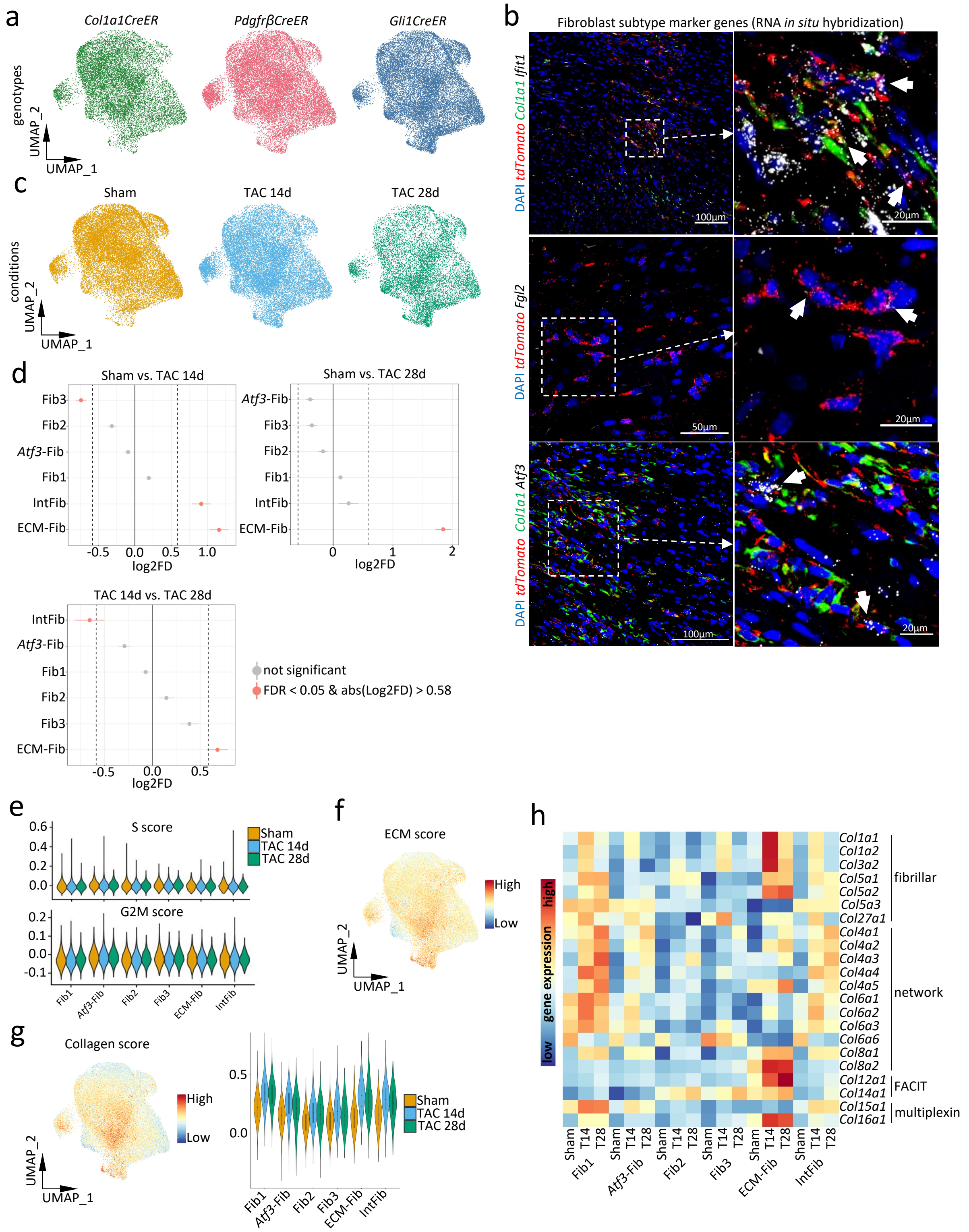

Supplementary Fig. 4: Comprehensive analysis of fibroblast subclustering

a, UMAP embedding of subclustered fibroblasts representing the underlying genotype of each cell. **b**, Representative images of RNA *in situ* hybridization for *tdTomato*, *Ifit1*, *Fgl2*, *Atf3* and *Col1a1* on *Gli1CreER;tdTomato* (top) and *PdgfrβCreER;tdTomato* (middle/bottom) hearts. Arrows indicated cells with co-expression of indicated genes. **c,** UMAP similar to (a) highlighting the underlying condition of each cell. **d**, Cell cluster proportion analysis by scProportionTest, testing for significant changes in cluster composition between the conditions sham and TAC (14 days & 28 days, mean ± 95% confidence interval). **e**, Violin plot of summarized G2M and S scores per condition and fibroblast subtype, based on the Seurat cell cycle scoring vignette. **f-g,** UMAP embedding of extracellular matrix (ECM) score (**f**) and collagen score (**g**) per cell of subclustered fibroblasts. On

the right, violin plot of collagen score summarized per subcluster and condition. Integrated boxplots show center line as median, box limits as upper and lower quartiles. Groups in violin plots were tested for significant difference between conditions (two-sided Wilcoxon rank sum test, unpaired, Bonferroni adjusted p-value). **h**, Heatmap of selected collagen subtype expression separated by fibroblast subtypes and conditions. Functional subtypes of collagens are indicated[2].

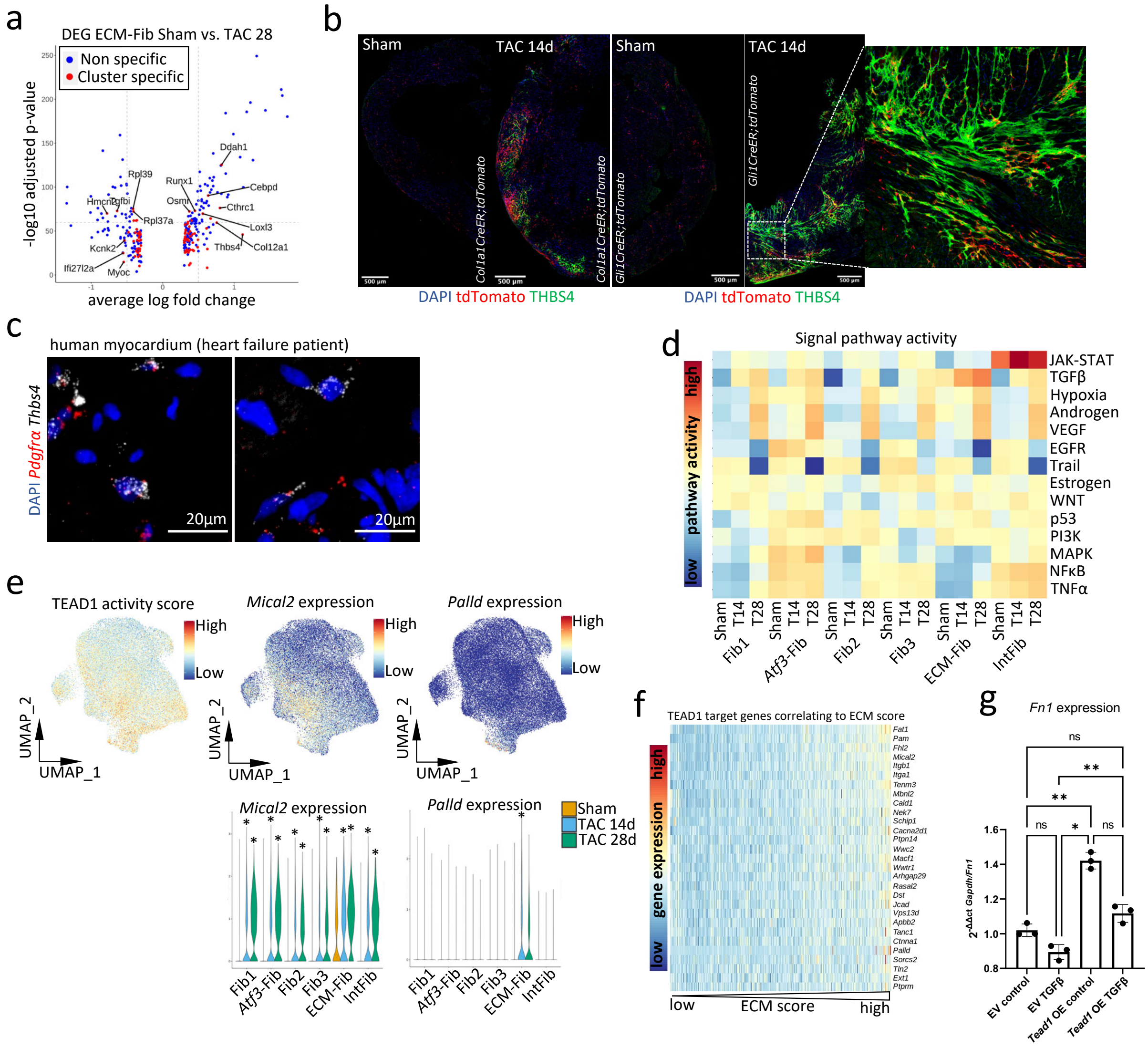

Supplementary Fig. 5: Transcription changes in fibroblasts and TEAD1 profibrotic relation

a, Differentially expressed genes (DEG) of ECM-Fib comparing sham and TAC 28 days visualized in volcano plot, displaying cluster specific (red) and non-specific (blue) DEG. **b,** Confocal immunofluorescence overview scans (10X objective) of *Col1a1CreER* and *Gli1CreER* tagged heart (sham/TAC) for indicated markers. For *Gli1CreER*, high magnifications of THBS4 area in TACs (bar 500µm). **c,** Representative pictures of RNA *in situ* hybridization for *Pdgfrα* and *Thbs4* on human heart failure patient myocardium. **d,** Signal pathway activity prediction of fibroblast subtypes based on pathway responsive genes (provided by PROGENy). Color indicates relative predicted activity per pathway. **e,** UMAP representation of TEAD1 activity score, *Mical2* and *Palld* expression of all fibroblasts. Color indicates level of expression or score. For *Mical2* and *Palld* violin plots of summarized expression between fibroblast subtypes and conditions are provided (*: adjusted p-value < 0.01, differential gene expression analysis by MAST). **f,** Heatmap of TEAD1 target gene expression correlating to high ECM expression in all fibroblasts. **g,** Bar graphs of relative gene expression of *Fn1* measured by RT-qPCR. Data points represent normalized expression by $2^{-\Delta\Delta Ct}$ method (mean ± SD, n = 3 independent experiments per group, *: p-value < 0.05; **: p-value < 0.01; ns: not significant; one-way ANOVA with Tukey's post-hoc). Source data are provided as a Source Data file.

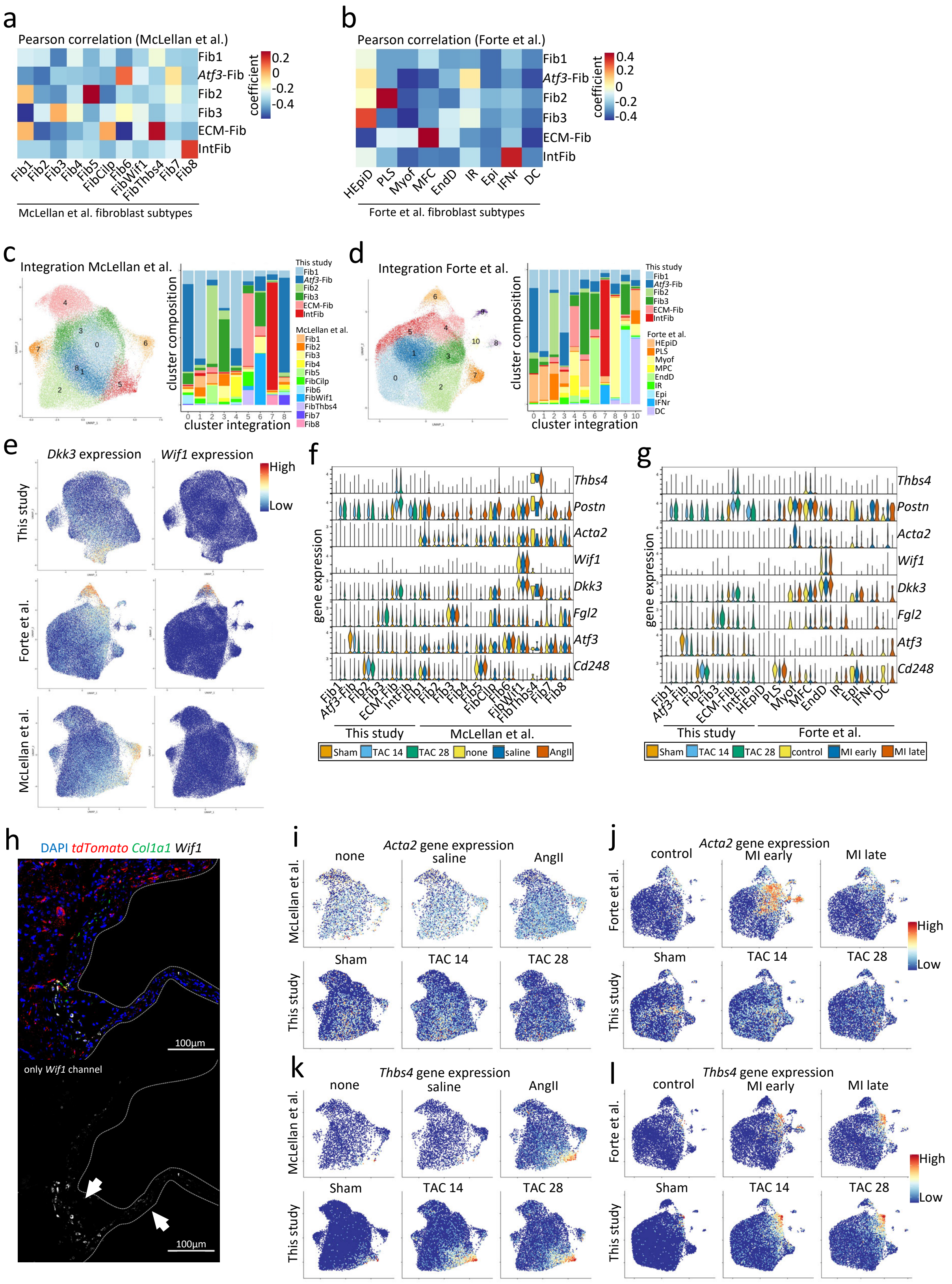

Supplementary Fig. 6: Fibroblast heterogeneity in different models of cardiac injury

a-b, Pearson correlation of fibroblast subclusters from this study and subclusters based on public available datasets from (a) McLellan et al. and (b) Forte et al.. Correlation was calculated on the 500 most variable genes, calculated by the FindVariableFeature function from Seurat. (homeostatic epicardial derived fibroblast - HEpiD, progenitor-like state fibroblast - PLS, myofibroblast - Myof, matrifibrocyte - MFC, endocardial-derived fibroblast - EndD, injury response fibroblast - IR, Epicaridum - Epi, interferon-response fibroblast - IFNr, dendritic-like - DC) **c-d**,

Integration and clustering (based on Seurat) of fibroblast from this study and fibroblast data from publicly available datasets from (c) McLellan et al. and (d) Forte et al.. UMAP embeddings show unsupervised clustering of the integrated fibroblasts. Bar plots show the cluster composition according to the previous annotation of the datasets without integration. **e**, UMAP of fibroblasts separated for each dataset (this study, Forte et al., McLellan et al.) showing gene expression for *Dkk3* and *Wif1*. **f-g**, Violin plots of selected marker gene expression from merged fibroblast datasets separated by conditions, (f) merged with McLellan et al., (g) merged with Forte et al.. **h**, Representative images of RNA *in situ* hybridization for *tdTomato*, *Wif1*and *Col1a1* on *Gli1CreER;tdTomato* heart tissue. The heart was cut at the longitudinal axis and the line highlights the aortic heart valve. Arrows indicate cells with *Wif1* expression. **i-l**, Multiple UMAP representation of the fibroblast dataset integrations as shown in (c-d). Gene expression of (i-j) *Acta2* and (k-l) *Thbs4* is shown separated by dataset and conditions.

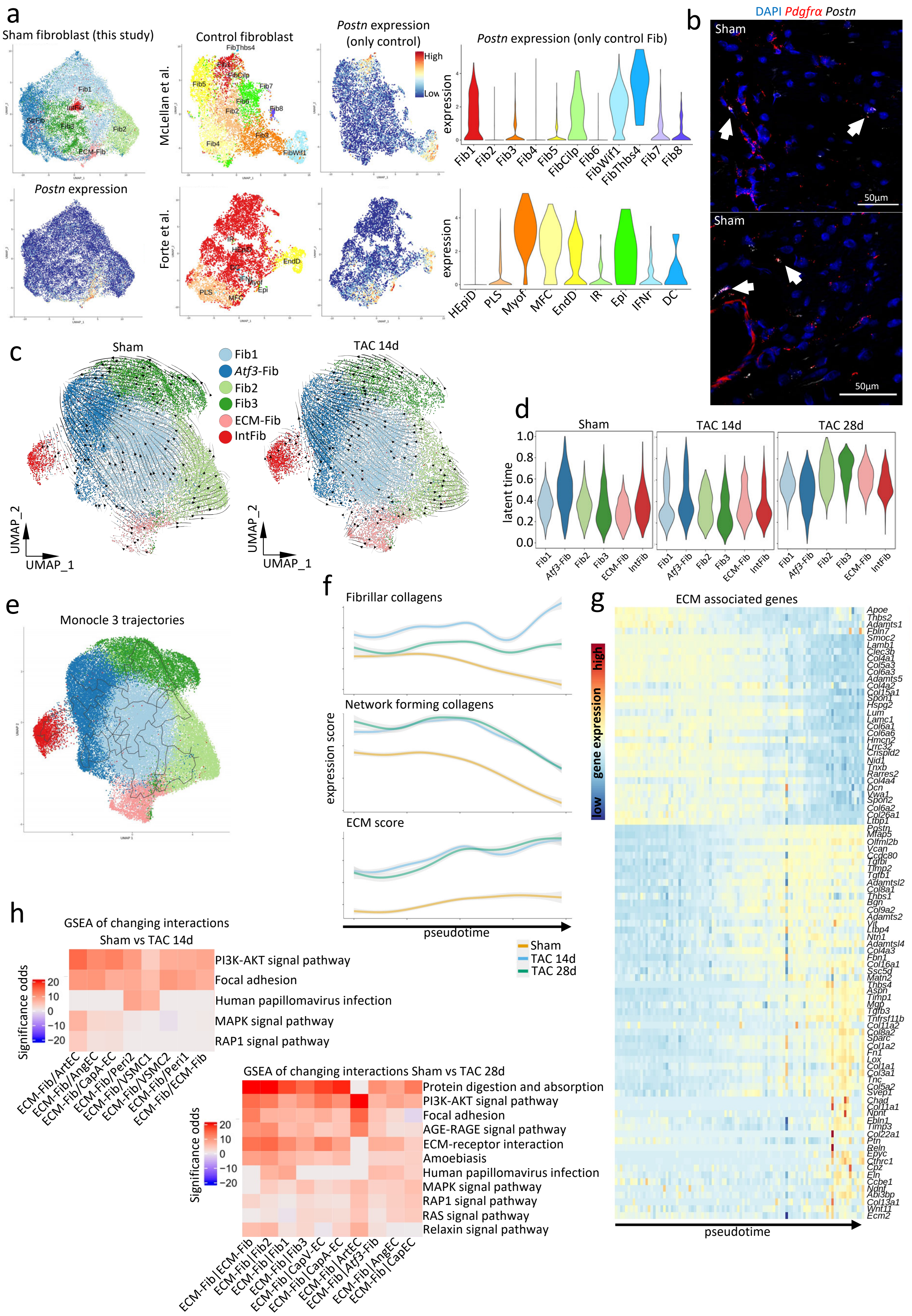

Supplementary Fig. 7: ECM-Fib origin and transcriptional switch of ECM expression

a, UMAP representation of the fibroblast control samples from this study (left), the McLellan et al. dataset and Forte et al. dataset (right). The respective control samples were integrated with Seurat and the annotations from

the full dataset were transferred. UMAPs show the gene expression for *Postn*. Violin plots show *Postn* gene expression of control fibroblasts summarized by transferred cluster (homeostatic epicardial derived fibroblast - HEpiD, progenitor-like state fibroblast - PLS, myofibroblast - Myof, matrifibrocyte - MFC, endocardial-derived fibroblast - EndD, injury response fibroblast - IR, Epicaridum - Epi, interferon-response fibroblast - IFNr, dendritic-like - DC). **b**, Representative images of RNA *in situ* hybridization for *Pdgfrα* and *Postn* in *PdgfrβCreER* murine sham hearts. Arrows indicate cells with co-expression of the stained markers. **c**, Velocities derived from the dynamical model for subclustered fibroblast (left, fibroblast from sham, right fibroblast from TAC 14d) are visualized as streamlines in a UMAP-based embedding. **d**, Violin plots of calculated latent time (from scVelo[3]) summarized per subcluster and separated by condition. Latent time is based on RNA velocities and shows a cell's transcriptional dynamics. **e**, UMAP of integrated fibroblasts with trajectories predicted by monocle 3. **f**, Smoothened scores fibrillar collagens, network forming collagens and ECM over pseudotime (x-axis) separated by condition: Sham, TAC 14 days and 28 days (gray error band: 95% confidence interval). **g**, Heatmap of gene expression for ECM associated genes, sorted by pseudotime on the x-axis. **h**, GSEA of changing interaction between sham and TAC involving ECM-Fib (upper left, sham vs. TAC 14 days, lower right sham vs. TAC 28 days). Color indicates up- or down significance odds.

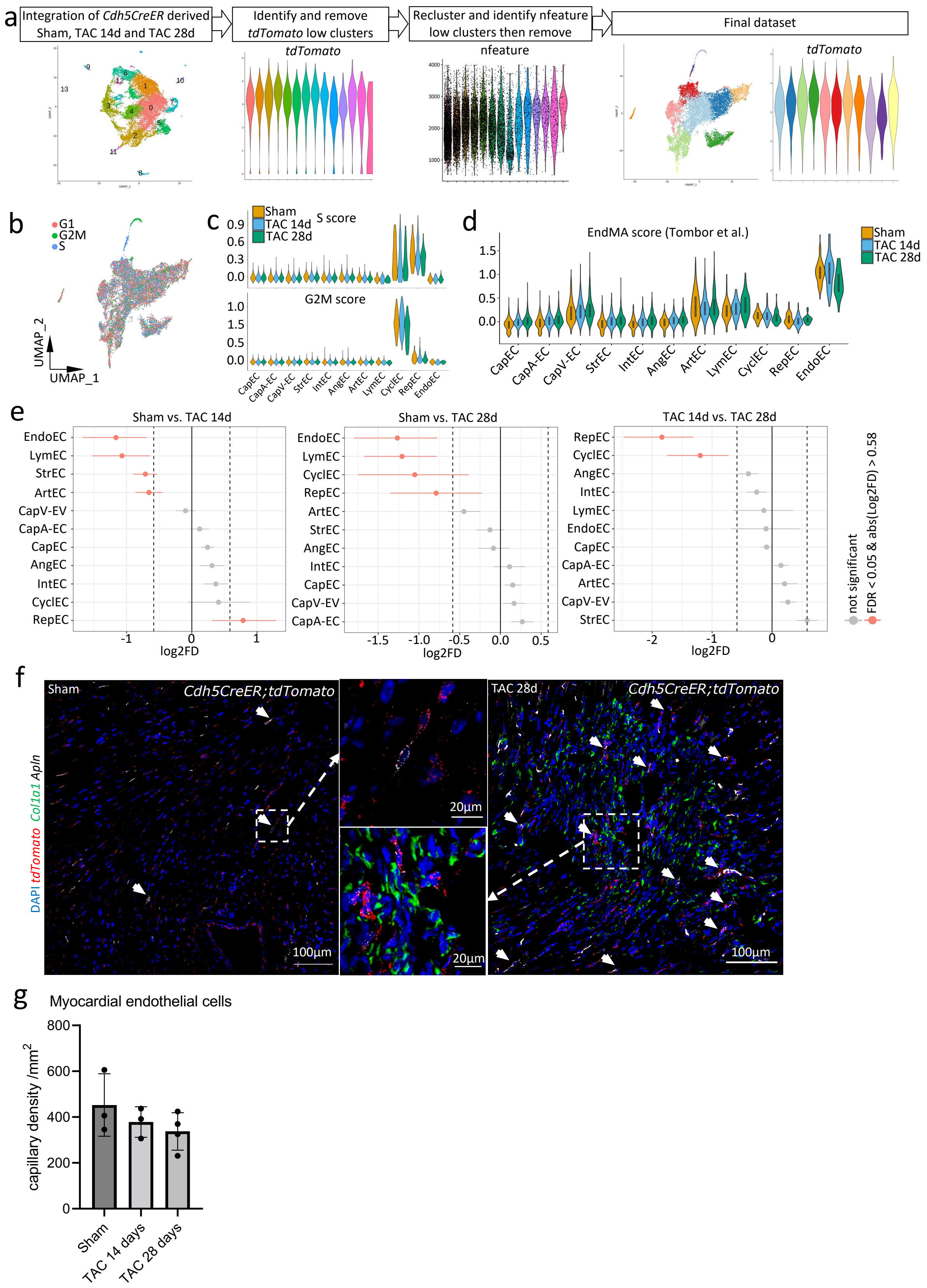

Supplementary Fig. 8. Data processing and subsequence analysis of the endothelial subtype integration
a, Stepwise representation of data processing and filtering of the integration of all samples contributing to the endothelial cell analysis. Only *Cdh5CrER* derived samples of sham and TAC14 days and 28 days were integrated pairwise using canonical correlation analysis from the Seurat package and filtered for clusters with low *tdTomato* expression and low feature count. **b**, UMAP embedding showing predicted cell cycle phase per cell, based on the

Seurat cell cycle scoring vignette. **c**, Violin plot of summarized G2M and S scores per condition and EC subtype, based on the Seurat cell cycle scoring vignette. **d**, Violin plot of summarized scores for transient mesenchymal activation (EndMA)[4] per condition and EC subcluster. Integrated boxplots show center line as median, box limits as upper and lower quartiles. **e**, Cellcluster proportion analysis by scProportionTest, testing for significant changes in cluster composition between the conditions sham and TAC (14 days & 28 days, mean ± 95% confidence interval). **f**, Representative images of RNA *in situ* hybridization for *Apln*, *tdTomato* and *Col1a1* on *Cdh5CreER;tdTomato* hearts. Left and upper middle picture show a sham heart, right and lower middle show a TAC heart. Arrows indicate cells with co-expression of *Apln* and *tdTomato.* **g**, Quantification of myocardial endothelial cells was performed by calculating the ratio of endothelial cells to myocardial area (mm^2). Image analysis was done in ImageJ (mean ± SD; n = 3 sham, n = 3 TAC 14d, n = 4 TAC 28d, each n represents an independent heart sample). Source data are provided as a Source Data file.

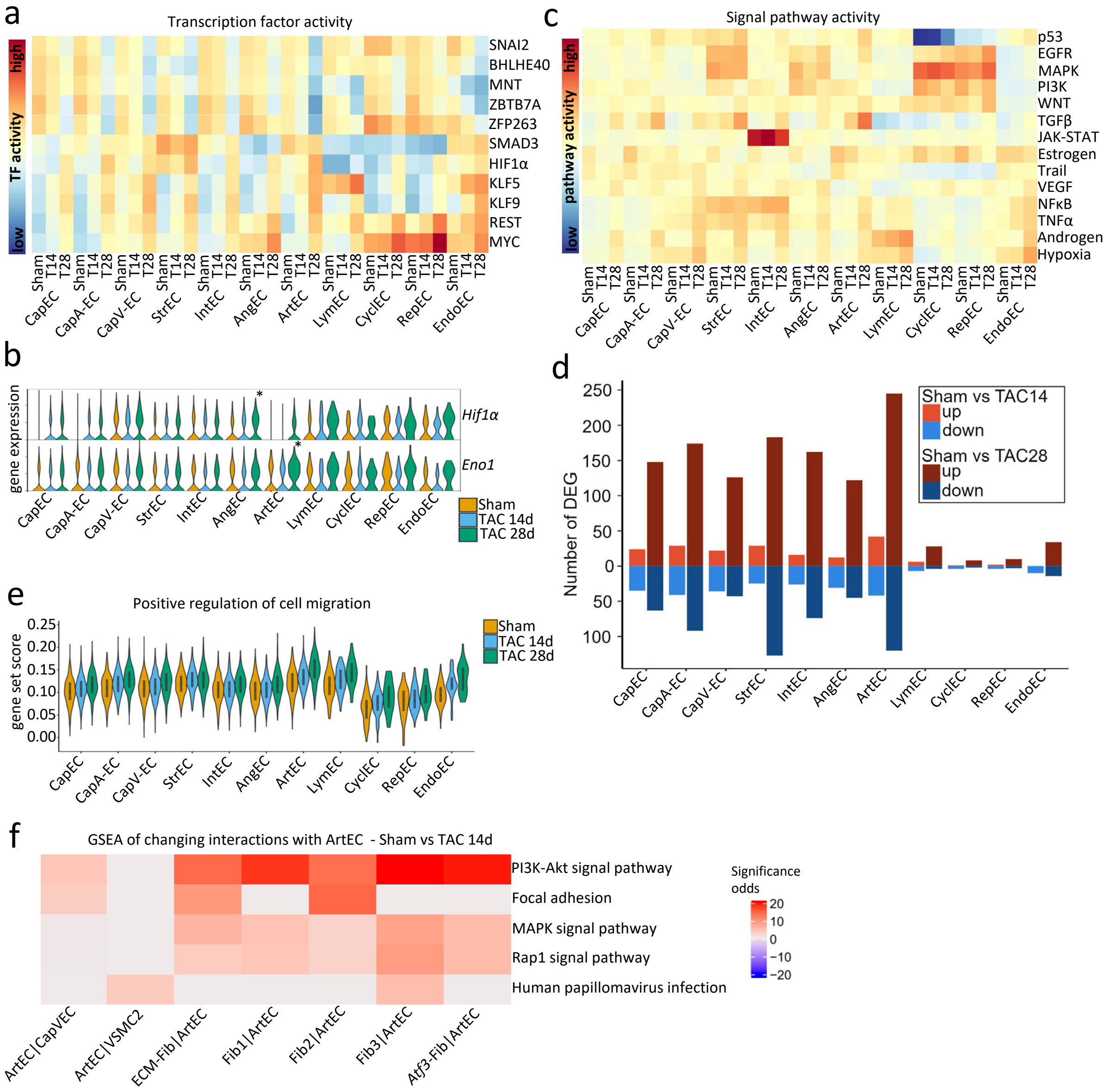

Supplementary Fig. 9 Specific transcriptomic and signaling changes in EC

a, TF activity prediction based on TF regulons (provided by DoRothEA) for EC subtypes. Color indicates relative predicted activity per TF. **b**, Violin plot of gene expression for *Hif1α* and *Eno1* per cluster split up into cells from either sham or TAC 14 days and TAC 28 days conditions (*: p-value < 0.001, differential gene expression analysis by MAST). **c**, Signal pathway activity prediction of EC subtypes based on pathway responsive genes (provided by PROGENy). Color indicates relative predicted activity per pathway. **d,** Bar plot of the number of differentially expressed genes (DEG) per subcluster, color indicates up and downregulation in TAC14/28 vs sham. **e,** Violin plot of summarized scores for positive regulation of cell migration per condition and EC cluster. Integrated boxplots show center line as median, box limits as upper and lower quartiles. ArtEC show significant differences to all other subtypes (two-sided Wilcoxon rank sum test, unpaired, Bonferroni adjusted p-value). **f,** GSEA of changing interaction between sham and TAC 14d involving ArtEC. Color indicates up- or down significance odds.

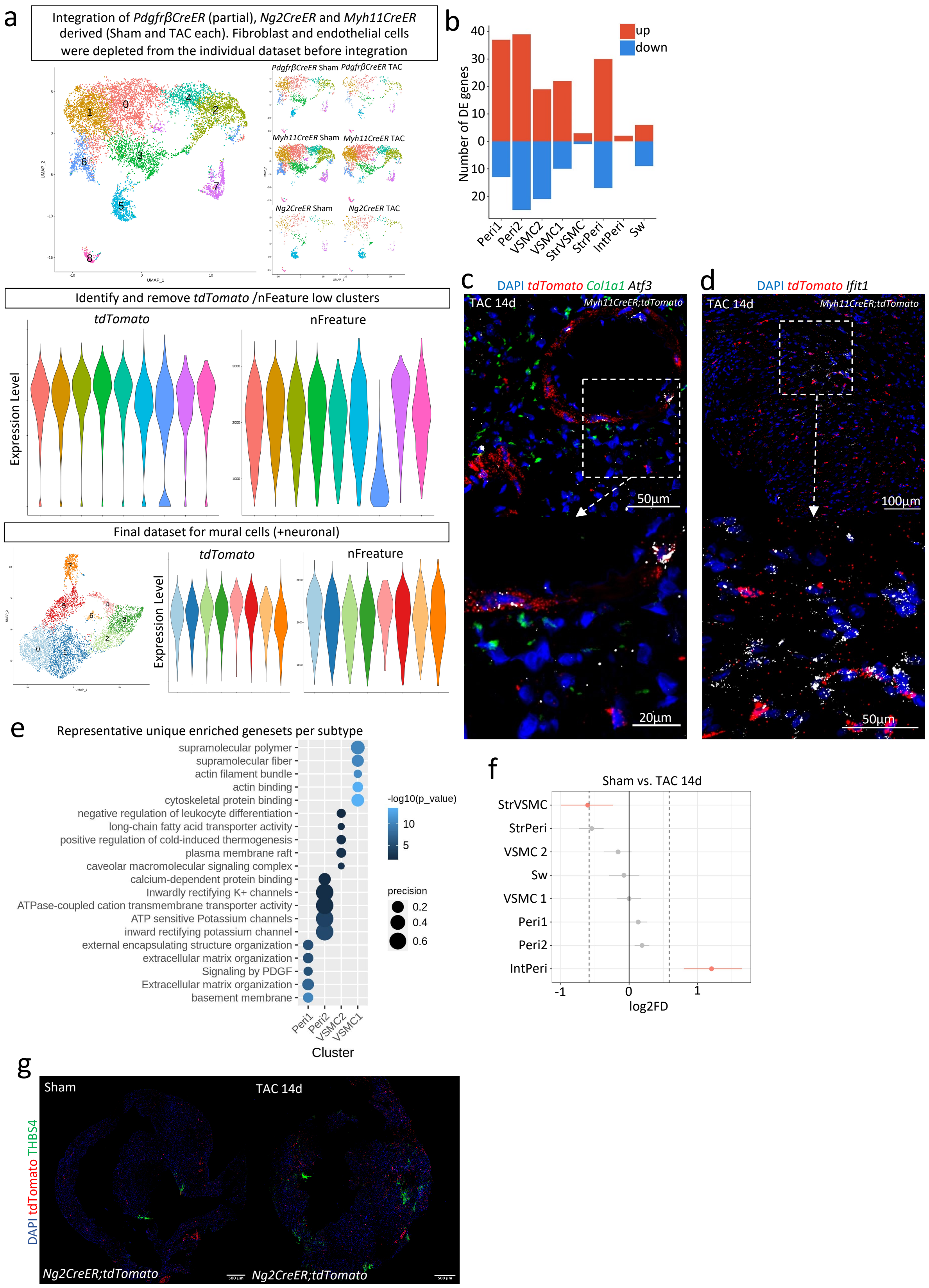

Supplementary Fig. 10: Data processing of the mural subtype integration

a, Stepwise representation of data processing and filtering of the integration of all samples contributing to the fibroblast analysis. Mural cells from the *Myh11CreER*, *Ng2CreER* and *PdgfrβCreER* lineage were integrated using canonical correlation analysis from the Seurat package and filtered for clusters with low *tdTomato* expression and low feature count. From the *PdgfrβCreER* datasets only cells identified as mural were included

for the integration. For the *Myh11CreER* datasets an additional filter step was required, where contaminating EC were depleted before integration. **b**, Bar plot of the number of differentially expressed genes (DEG) comparing TAC 14 days vs sham per mural cell subcluster. **c**, Representative images of RNA *in situ* hybridization for *Atf3*, *tdTomato* and *Col1a1* on *Myh11CreER;tdtomato* hearts. **d**, Representative images of RNA *in situ* hybridization for *Ifit1*, *tdTomato* and *Col1a1* on *Myh11CreER;tdTomato* hearts. **e**, Top five representative gene sets (hypergeometric test, one-sided) uniquely enriched for each subcluster, based on marker genes per mural cell subtype. Dot size refers to overlap of tested genes and gene set (precision). **f**, Cell cluster proportion analysis by scProportionTest, testing for significant changes in cluster composition between the conditions sham and TAC (mean ± 95% confidence interval). **g**, Confocal immunofluorescence overview scans of *Ng2CreER* tagged hearts (sham/TAC) stained for THBS4 (bar 500μm). For details on statistics and reproducibility, see Methods.

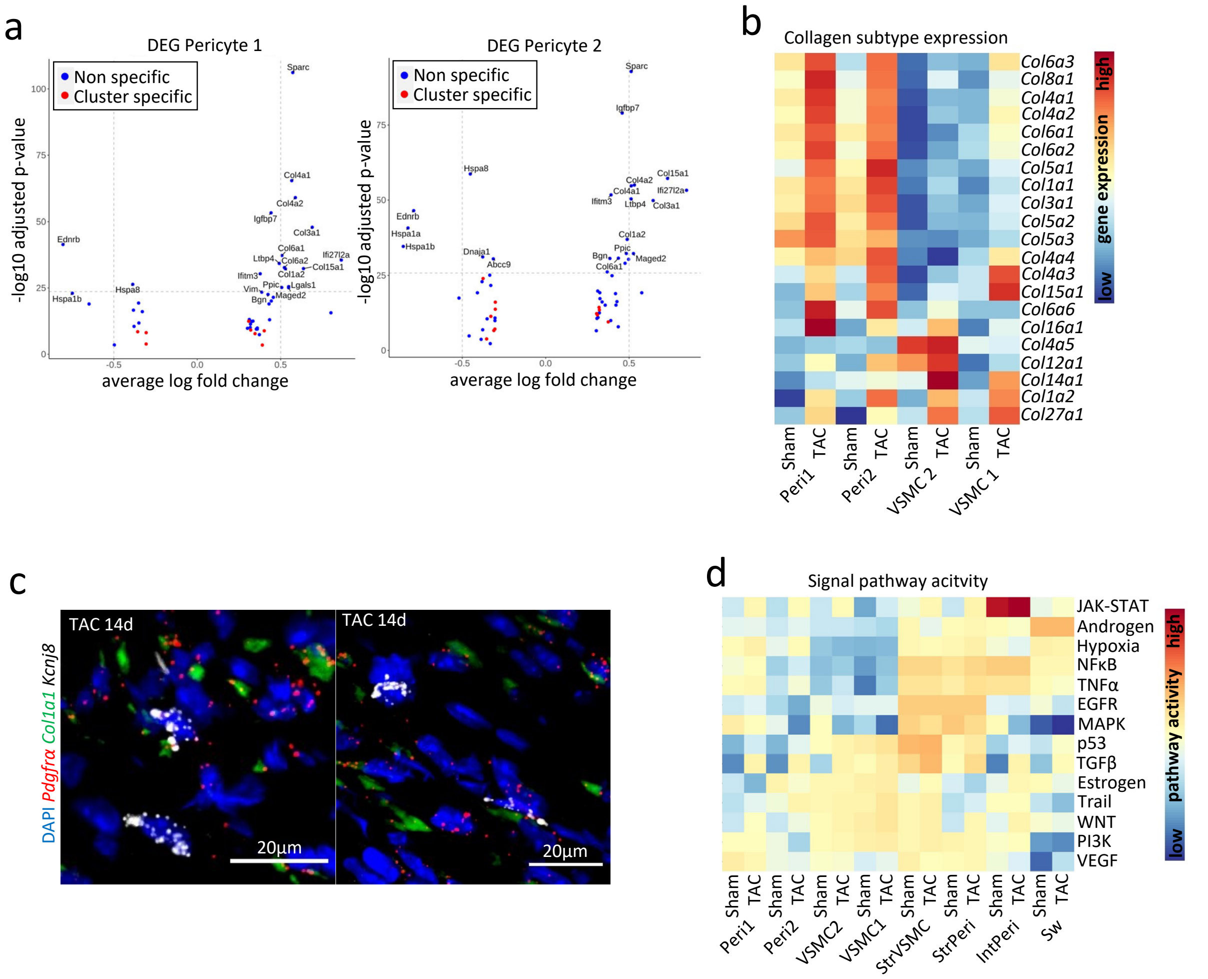

Supplementary Fig. 11: Subsequent analysis of mural subtype integration

a, Differentially expressed genes (DEG) of Peri1 and Peri2 comparing sham and TAC visualized in volcano plots, displaying cluster specific (red) and non-specific (blue) DEG. **b**, Heatmap of collagen subtype expression in selected mural cell subclusters separated by condition. **c**, Representative images of RNA *in situ* hybridization for *Kcnj8, Pdgfrα* and *Col1a1* on hearts after TAC. **d**, Signal pathway activity prediction of mural cell subtypes based on pathway responsive genes (provided by PROGENy). Color indicates relative predicted activity per pathway.

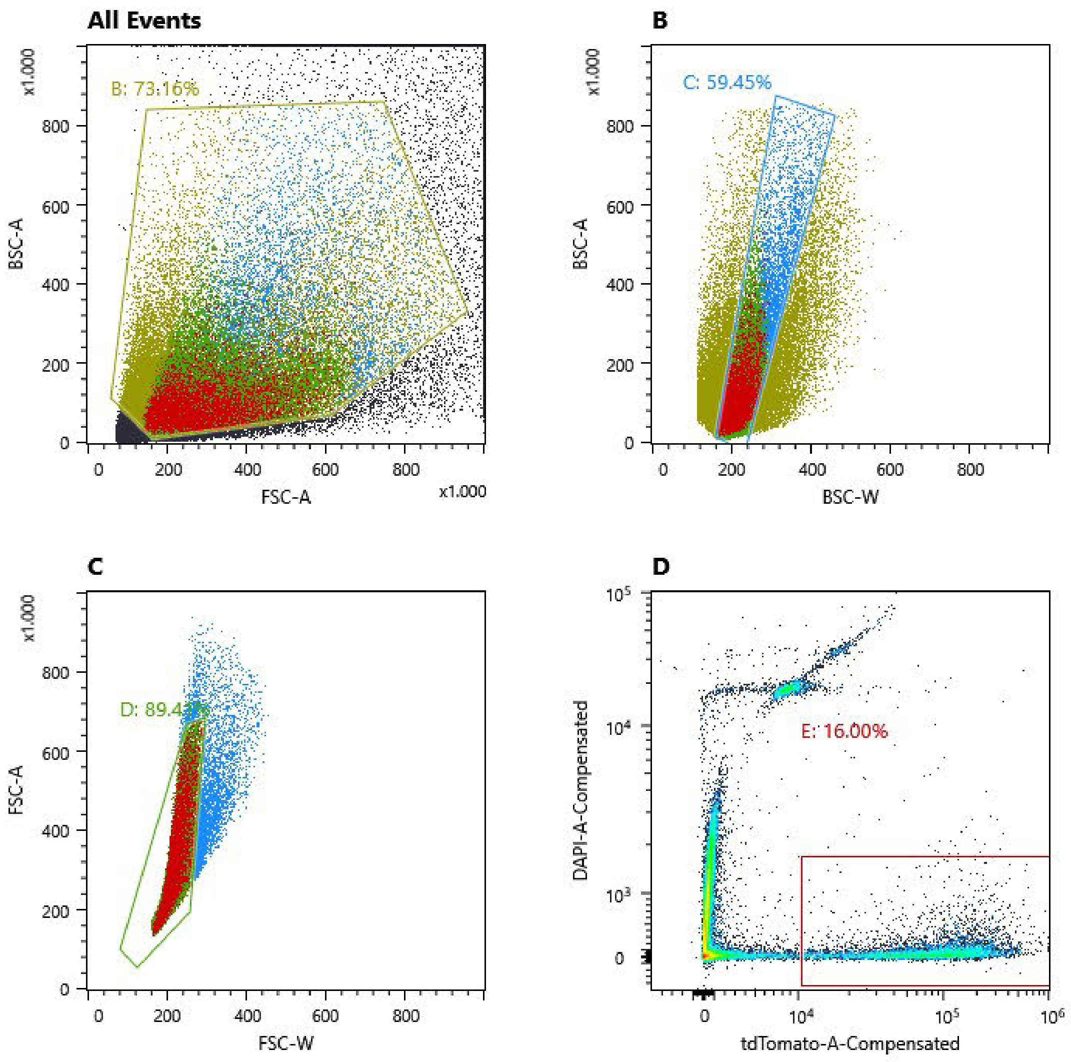

Supplementary Fig. 12: FACS gating strategy for sorting tdTomato expressing fate traced cells
Representative flow cytometric plots from the sorting and gating strategy used to sort for tdTomato expression fate traced cardiac cells.

Supplementary Table 1: *tdTomato* gene sequence added to reference genome mm10

***tdTomato* sequence**
ATAACTTCGTATAATGTATGCTATACGAAGTTATTAGGTCCCTCGACCTGCAGCCCAAGCT<br>AGATCGAATTCGGCCGGCCTTGTACGCGTTAAGTGCAACACGATCCCGCCACCATGGTGA<br>GCAAGGGCGAGGAGGTCATCAAAGAGTTCATGCGCTTCAAGGTGCGCATGGAGGGCTCC<br>ATGAACGGCCACGAGTTCGAGATCGAGGGCGAGGGCGAGGGCCGCCCCTACGAGGGCAC<br>CCAGACCGCCAAGCTGAAGGTGACCAAGGGCGGCCCCCTGCCCTTCGCCTGGGACATCCT<br>GTCCCCCCAGTTCATGTACGGCTCCAAGGCGTACGTGAAGCACCCCGCCGACATCCCCGA<br>TTACAAGAAGCTGTCCTTCCCCGAGGGCTTCAAGTGGGAGCGCGTGATGAACTTCGAGGA<br>CGGCGGTCTGGTGACCGTGACCCAGGACTCCTCCCTGCAGGACGGCACGCTGATCTACAA<br>GGTGAAGATGCGCGGCACCAACTTCCCCCCCGACGGCCCCGTAATGCAGAAGAAGACCA<br>TGGGCTGGGAGGCCTCCACCGAGCGCCTGTACCCCCGCGACGGCGTGCTGAAGGGCGAG<br>ATCCACCAGGCCCTGAAGCTGAAGGACGGCGGCCACTACCTGGTGGAGTTCAAGACCAT<br>CTACATGGCCAAGAAGCCCGTGCAACTGCCCGGCTACTACTACGTGGACACCAAGCTGGA<br>CATCACCTCCCACAACGAGGACTACACCATCGTGGAACAGTACGAGCGCTCCGAGGGCC<br>GCCACCACCTGTTCCTGGGGCATGGCACCGGCAGCACCGGCAGCGGCAGCTCCGGCACC<br>GCCTCCTCCGAGGACAACAACATGGCCGTCATCAAAGAGTTCATGCGCTTCAAGGTGCGC<br>ATGGAGGGCTCCATGAACGGCCACGAGTTCGAGATCGAGGGCGAGGGCGAGGGCCGCCC<br>CTACGAGGGCACCCAGACCGCCAAGCTGAAGGTGACCAAGGGCGGCCCCCTGCCCTTCG<br>CCTGGGACATCCTGTCCCCCCAGTTCATGTACGGCTCCAAGGCGTACGTGAAGCACCCCG<br>CCGACATCCCCGATTACAAGAAGCTGTCCTTCCCCGAGGGCTTCAAGTGGGAGCGCGTGA<br>TGAACTTCGAGGACGGCGGTCTGGTGACCGTGACCCAGGACTCCTCCCTGCAGGACGGCA<br>CGCTGATCTACAAGGTGAAGATGCGCGGCACCAACTTCCCCCCCGACGGCCCCGTAATGC<br>AGAAGAAGACCATGGGCTGGGAGGCCTCCACCGAGCGCCTGTACCCCCGCGACGGCGTG<br>CTGAAGGGCGAGATCCACCAGGCCCTGAAGCTGAAGGACGGCGGCCACTACCTGGTGGA<br>GTTCAAGACCATCTACATGGCCAAGAAGCCCGTGCAACTGCCCGGCTACTACTACGTGGA<br>CACCAAGCTGGACATCACCTCCCACAACGAGGACTACACCATCGTGGAACAGTACGAGC<br>GCTCCGAGGGCCGCCACCACCTGTTCCTGTACGGCATGGACGAGCTGTACAAGTAAGAAT<br>TGTGTTGCACTTAACGCGTACAAGGCCGGCCCTGCAGGAATTCGATATCAAGCTTATCGA<br>TAATCAACCTCTGGATTACAAAATTTGTGAAAGATTGACTGGTATTCTTAACTATGTTGCT<br>CCTTTTACGCTATGTGGATACGCTGCTTTAATGCCTTTGTATCATGCTATTGCTTCCCGTAT<br>GGCTTTCATTTTCTCCTCCTTGTATAAATCCTGGTTGCTGTCTCTTTATGAGGAGTTGTGGC<br>CCGTTGTCAGGCAACGTGGCGTGGTGTGCACTGTGTTTGCTGACGCAACCCCCACTGGTT<br>GGGGCATTGCCACCACCTGTCAGCTCCTTTCCGGGACTTTCGCTTTCCCCCTCCCTATTGC<br>CACGGCGGAACTCATCGCCGCCTGCCTTGCCCGCTGCTGGACAGGGGCTCGGCTGTTGGG<br>CACTGACAATTCCGTGGTGTTGTCGGGGAAATCATCGTCCTTTCCTTGGCTGCTCGCCTGT<br>GTTGCCACCTGGATTCTGCGCGGGACGTCCTTCTGCTACGTCCCTTCGGCCCTCAATCCAG<br>CGGACCTTCCTTCCCGCGGCCTGCTGCCGGCTCTGCGGCCTCTTCCGCGTCTTCGCCTTCG<br>CCCTCAGACGAGTCGGATCTCCCTTTGGGCCGCCTCCCCGCATCGATACCGTCGACCTCG<br>ACCT

Supplementary Table 2: R packages info

attached packages and version	loaded via a namespace (+version)	loaded via a namespace (+version)
reticulate_1.18	tidyselect_1.1.0	rio_0.5.16
enrichR_3.0	htmlwidgets_1.5.2	mclust_5.4.7
KEGGREST_1.28.0	grid_4.0.2	gridExtra_2.3
readxl_1.3.1	Rtsne_0.15	compiler_4.0.2
svglite_1.2.3.2	munsell_0.5.0	maps_3.3.0
ggrepel_0.8.2	codetools_0.2-16	KernSmooth_2.23-17
progeny_1.10.0	ica_1.0-2	crayon_1.3.4
viridis_0.5.1	future_1.20.1	htmltools_0.5.1.1
viridisLite_0.3.0	miniUI_0.1.1.1	segmented_1.3-0
gprofiler2_0.2.0	withr_2.3.0	mgcv_1.8-33
SeuratWrappers_0.3.0	colorspace_2.0-0	later_1.1.0.1
monocle3_0.2.3.0	rstudioapi_0.13	tweenr_1.0.1
SingleCellExperiment_1.12.0	ROCR_1.0-11	MASS_7.3-53.1
GenomicRanges_1.42.0	ggsignif_0.6.0	bcellViper_1.24.0
GenomeInfoDb_1.26.1	tensor_1.5	car_3.0-10
IRanges_2.24.0	listenv_0.8.0	igraph_1.2.6
S4Vectors_0.28.0	GenomeInfoDbData_1.2.4	forcats_0.5.0
MatrixGenerics_1.2.0	polyclip_1.10-0	pkgconfig_2.0.3
matrixStats_0.58.0	farver_2.0.3	foreign_0.8-80
scProportionTest_0.0.0.9000	parallelly_1.21.0	plotly_4.9.2.1
colorBlindness_0.1.6	vctrs_0.3.5	XVector_0.30.0
scales_1.1.1	generics_0.1.0	digest_0.6.27
RColorBrewer_1.1-2	R6_2.5.0	sctransform_0.3.1
harmony_1.0	graphlayouts_0.7.1	RcppAnnoy_0.0.17
Rcpp_1.0.6	rsvd_1.0.3	Biostrings_2.56.0
pals_1.6	locfit_1.5-9.4	spatstat.data_2.1-0
stringr_1.4.0	bitops_1.0-6	cellranger_1.1.0
reshape2_1.4.4	spatstat.utils_2.1-0	leiden_0.3.5
dittoSeq_1.0.2	gridGraphics_0.5-0	uwot_0.1.9
ggpubr_0.4.0	DelayedArray_0.16.0	edgeR_3.32.0
writexl_1.3.1	promises_1.1.1	gdtools_0.2.2
genesorteR_0.4.3	gtable_0.3.0	curl_4.3
Matrix_1.3-2	globals_0.14.0	kernlab_0.9-29
clustree_0.4.3	goftest_1.2-2	shiny_1.5.0
ggraph_2.0.4	tidygraph_1.2.0	rjson_0.2.20
ggplot2_3.3.3	rlang_0.4.12	lifecycle_0.2.0
cowplot_1.1.0	systemfonts_0.3.2	nlme_3.1-150
Seurat_3.2.2	splines_4.0.2	jsonlite_1.7.1
readr_1.4.0	rstatix_0.6.0	carData_3.0-4
pheatmap_1.0.12	lazyeval_0.2.2	mapproj_1.2.7
tidyr_1.1.2	dichromat_2.0-0	limma_3.46.0
tibble_3.0.4	broom_0.7.2	pillar_1.4.7
dplyr_1.0.2	BiocManager_1.30.10	lattice_0.20-41
viper_1.22.0	abind_1.4-5	fastmap_1.0.1
Biobase_2.50.0	backports_1.2.0	httr_1.4.2
BiocGenerics_0.36.0	httpuv_1.5.4	survival_3.2-7
dorothea_1.3.0	tools_4.0.2	glue_1.4.2
	ellipsis_0.3.1	remotes_2.2.0
	ggridges_0.5.2	zip_2.1.1
	plyr_1.8.6	spatstat_1.64-1
	zlibbioc_1.36.0	png_0.1-7
	purrr_0.3.4	ggforce_0.3.2
	RCurl_1.98-1.2	class_7.3-17
	rpart_4.1-15	stringi_1.5.3
	deldir_0.2-3	mixtools_1.2.0
	pbapply_1.4-3	irlba_2.3.3
	zoo_1.8-8	e1071_1.7-4
	haven_2.3.1	future.apply_1.6.0
	cluster_2.1.0	
	magrittr_2.0.1	
	data.table_1.14.0	
	openxlsx_4.2.3	
	lmtest_0.9-38	
	RANN_2.6.1	
	fitdistrplus_1.1-3	
	hms_0.5.3	
	patchwork_1.1.0	
	mime_0.9	
	xtable_1.8-4	

References:

1. Richards, D. A. *et al.* Distinct Phenotypes Induced by Three Degrees of Transverse Aortic Constriction in Mice. *Sci. Rep.* **9**, 5844 (2019).
2. Karsdal, M. A. *et al.* Collagen biology and non-invasive biomarkers of liver fibrosis. *Liver Int.* **40**, 736–750 (2020).
3. Bergen, V., Lange, M., Peidli, S., Alexander Wolf, F. & Theis, F. J. Generalizing RNA velocity to transient cell states through dynamical modeling. *Nat. Biotechnol.* **38**, 1408–1414 (2020).
4. Tombor, L. S. *et al.* Single cell sequencing reveals endothelial plasticity with transient mesenchymal activation after myocardial infarction. *Nat. Commun.* **12**, 681 (2021).
