# Supplementary material for: Mapping the cardiac vascular niche in heart failure
Source: Nat Commun. 2022 May 31;13:3027. doi: 10.1038/s41467-022-30682-0 (PMC9156759; doi:10.1038/s41467-022-30682-0)
Supplement: Supplementary file 3 — Description of Additional Supplementary Files [file 41467_2022_30682_MOESM3_ESM.docx]

Description of Additional Supplementary Files

File Name: Supplementary Data 1
Description: This file contains the results of the gene set enrichment analysis based on cluster specific marker genes. Enriched gene sets are filtered for the following cell types: fibroblasts, mural cells, and endothelial cells.
