# Supplementary material for: Mapping the cardiac vascular niche in heart failure
Source: Nat Commun. 2022 May 31;13:3027. doi: 10.1038/s41467-022-30682-0 (PMC9156759; doi:10.1038/s41467-022-30682-0)
Supplement: Supplementary file 5 — Reporting Summary [file 41467_2022_30682_MOESM5_ESM.pdf]

nature portfolio

Corresponding author(s): R. Kramann

Last updated by author(s): April 14, 2022

Reporting Summary

Nature Portfolio wishes to improve the reproducibility of the work that we publish. This form provides structure for consistency and transparency in reporting. For further information on Nature Portfolio policies, see our Editorial Policies and the Editorial Policy Checklist.

Statistics

For all statistical analyses, confirm that the following items are present in the figure legend, table legend, main text, or Methods section.

n/a	Confirmed	
☐	☒	The exact sample size (*n*) for each experimental group/condition, given as a discrete number and unit of measurement
☐	☒	A statement on whether measurements were taken from distinct samples or whether the same sample was measured repeatedly
☐	☒	The statistical test(s) used AND whether they are one- or two-sided<br>*Only common tests should be described solely by name; describe more complex techniques in the Methods section.*
☐	☒	A description of all covariates tested
☐	☒	A description of any assumptions or corrections, such as tests of normality and adjustment for multiple comparisons
☐	☒	A full description of the statistical parameters including central tendency (e.g. means) or other basic estimates (e.g. regression coefficient) AND variation (e.g. standard deviation) or associated estimates of uncertainty (e.g. confidence intervals)
☐	☒	For null hypothesis testing, the test statistic (e.g. *F*, *t*, *r*) with confidence intervals, effect sizes, degrees of freedom and *P* value noted<br>*Give P values as exact values whenever suitable.*
☒	☐	For Bayesian analysis, information on the choice of priors and Markov chain Monte Carlo settings
☒	☐	For hierarchical and complex designs, identification of the appropriate level for tests and full reporting of outcomes
☐	☒	Estimates of effect sizes (e.g. Cohen's *d*, Pearson's *r*), indicating how they were calculated

Our web collection on statistics for biologists contains articles on many of the points above.

Software and code

Policy information about availability of computer code

Data collection	Sony FACS software (Version 2.1.6) was used for cell sorting on the Sony SH800.<br>Fluorescent and brightfield images were acquired using Nikon software (NIS-Elements AR 5.11.03) on<br>Nikon A1R confocal microscope using 40X and 60X objectives (Nikon). Brightfield images were taken by<br>brightfield microscopy (Leica) and analyzed using ImageJ (National Institutes of Health, Bethesda, MD).<br>Other Software used: R (Version 4.0.2), Python3 (Version 3.6.8), Cell Ranger (Version 3.0.2), Graphpad (Version 9.0.1),<br>ImageJ (1.53), CellPhoneDB (Version 2.1.1), Vevo Lab (Version 5.5.1).<br>Sequencing was performed on the NovaSeq Illumina sequencers at Rotterdam University Hospital respectively.
Data analysis	All R and Python packages used in data analysis are described here:<br>https://github.com/KramannLab/Murine_heart_map/references/r_requirements.txt<br>Additional software used for data analysis: Graphpad, ImageJ.<br>All scripts used for single cell RNA-seq data analysis are available here:<br>https://github.com/KramannLab/Murine_heart_map/scripts/<br>Other software used in data analysis Cell Ranger (v3.0.2), CellPhoneDB (v2.1.1).

For manuscripts utilizing custom algorithms or software that are central to the research but not yet described in published literature, software must be made available to editors and reviewers. We strongly encourage code deposition in a community repository (e.g. GitHub). See the Nature Portfolio guidelines for submitting code & software for further information.

Data

Policy information about availability of data

All manuscripts must include a data availability statement. This statement should provide the following information, where applicable:

- Accession codes, unique identifiers, or web links for publicly available datasets
- A description of any restrictions on data availability
- For clinical datasets or third party data, please ensure that the statement adheres to our policy

Processed and raw single cell RNA sequencing data is available via the Gene Expression Omnibus under the series number GSE166403. The reviewer token is ebmragiajjohher .

Field-specific reporting

Please select the one below that is the best fit for your research. If you are not sure, read the appropriate sections before making your selection.

☒ Life sciences ☐ Behavioural & social sciences ☐ Ecological, evolutionary & environmental sciences

For a reference copy of the document with all sections, see nature.com/documents/nr-reporting-summary-flat.pdf

Life sciences study design

All studies must disclose on these points even when the disclosure is negative.

Sample size	In total we present 14 scRNA-Seq libraries from 3-5 mice per library. No statistical methods were used to predetermine sample size due to the nature of this study. For all experiments we used the minimum number of animals needed to reliably detect the expected effect size with an alpha rate set at 0.05 in a standard powered experiment and based on extensive laboratory experience and literature in the field.
Data exclusions	For full details see description in Methods. For scRNA-Seq experiments, low-quality droplets were excluded based on transcript count, mitochondrial transcript content and tdTomato reads.<br>These exclusion criteria were pre-established prior to study data analysis.
Replication	Heart tissue form a minimum of 3 mice per library were pooled for RNA sequencing.<br>Immunofluorescenct imaging was performed on 3 heart samples minimum and repeated with similar results successfully.<br>In vitro experiments were repeated 3 times independent. Positive and negative controls were done once per used sample. We confirm that all attemps at replication were succesful.
Randomization	Mice were randomly allocated in the three different surgery groups in our study. Randomization for human tissue was not relevant due to validatory nature of the experiment.
Blinding	Experimenters were blinded during imaging. Tissue collections for gene expression were not performed blind given that subsequent experiments were carried by the same researcherrs from the beginning.

Behavioural & social sciences study design

All studies must disclose on these points even when the disclosure is negative.

Study description	*Briefly describe the study type including whether data are quantitative, qualitative, or mixed-methods (e.g. qualitative cross-sectional, quantitative experimental, mixed-methods case study).*
Research sample	*State the research sample (e.g. Harvard university undergraduates, villagers in rural India) and provide relevant demographic information (e.g. age, sex) and indicate whether the sample is representative. Provide a rationale for the study sample chosen. For studies involving existing datasets, please describe the dataset and source.*
Sampling strategy	*Describe the sampling procedure (e.g. random, snowball, stratified, convenience). Describe the statistical methods that were used to predetermine sample size OR if no sample-size calculation was performed, describe how sample sizes were chosen and provide a rationale for why these sample sizes are sufficient. For qualitative data, please indicate whether data saturation was considered, and what criteria were used to decide that no further sampling was needed.*
Data collection	*Provide details about the data collection procedure, including the instruments or devices used to record the data (e.g. pen and paper, computer, eye tracker, video or audio equipment) whether anyone was present besides the participant(s) and the researcher, and whether the researcher was blind to experimental condition and/or the study hypothesis during data collection.*
Timing	*Indicate the start and stop dates of data collection. If there is a gap between collection periods, state the dates for each sample cohort.*

Data exclusions	*If no data were excluded from the analyses, state so OR if data were excluded, provide the exact number of exclusions and the rationale behind them, indicating whether exclusion criteria were pre-established.*
Non-participation	*State how many participants dropped out/declined participation and the reason(s) given OR provide response rate OR state that no participants dropped out/declined participation.*
Randomization	*If participants were not allocated into experimental groups, state so OR describe how participants were allocated to groups, and if allocation was not random, describe how covariates were controlled.*

Ecological, evolutionary & environmental sciences study design

All studies must disclose on these points even when the disclosure is negative.

Study description	*Briefly describe the study. For quantitative data include treatment factors and interactions, design structure (e.g. factorial, nested, hierarchical), nature and number of experimental units and replicates.*
Research sample	*Describe the research sample (e.g. a group of tagged Passer domesticus, all Stenocereus thurberi within Organ Pipe Cactus National Monument), and provide a rationale for the sample choice. When relevant, describe the organism taxa, source, sex, age range and any manipulations. State what population the sample is meant to represent when applicable. For studies involving existing datasets, describe the data and its source.*
Sampling strategy	*Note the sampling procedure. Describe the statistical methods that were used to predetermine sample size OR if no sample-size calculation was performed, describe how sample sizes were chosen and provide a rationale for why these sample sizes are sufficient.*
Data collection	*Describe the data collection procedure, including who recorded the data and how.*
Timing and spatial scale	*Indicate the start and stop dates of data collection, noting the frequency and periodicity of sampling and providing a rationale for these choices. If there is a gap between collection periods, state the dates for each sample cohort. Specify the spatial scale from which the data are taken*
Data exclusions	*If no data were excluded from the analyses, state so OR if data were excluded, describe the exclusions and the rationale behind them, indicating whether exclusion criteria were pre-established.*
Reproducibility	*Describe the measures taken to verify the reproducibility of experimental findings. For each experiment, note whether any attempts to repeat the experiment failed OR state that all attempts to repeat the experiment were successful.*
Randomization	*Describe how samples/organisms/participants were allocated into groups. If allocation was not random, describe how covariates were controlled. If this is not relevant to your study, explain why.*
Blinding	*Describe the extent of blinding used during data acquisition and analysis. If blinding was not possible, describe why OR explain why blinding was not relevant to your study.*

Did the study involve field work? ☐ Yes ☐ No

Field work, collection and transport

Field conditions	*Describe the study conditions for field work, providing relevant parameters (e.g. temperature, rainfall).*
Location	*State the location of the sampling or experiment, providing relevant parameters (e.g. latitude and longitude, elevation, water depth).*
Access & import/export	*Describe the efforts you have made to access habitats and to collect and import/export your samples in a responsible manner and in compliance with local, national and international laws, noting any permits that were obtained (give the name of the issuing authority, the date of issue, and any identifying information).*
Disturbance	*Describe any disturbance caused by the study and how it was minimized.*

Reporting for specific materials, systems and methods

We require information from authors about some types of materials, experimental systems and methods used in many studies. Here, indicate whether each material, system or method listed is relevant to your study. If you are not sure if a list item applies to your research, read the appropriate section before selecting a response.

Materials & experimental systems

n/a	Involved in the study
☐	☒ Antibodies
☐	☒ Eukaryotic cell lines
☒	☐ Palaeontology and archaeology
☐	☒ Animals and other organisms
☐	☒ Human research participants
☒	☐ Clinical data
☒	☐ Dual use research of concern

Methods

n/a	Involved in the study
☒	☐ ChIP-seq
☐	☒ Flow cytometry
☒	☐ MRI-based neuroimaging

Antibodies

Antibodies used

Anti-Mouse CD31 (553370, 1:100, BD Biosciences)
Thrombospondin-4 Antibody (893655, 1:100, Novus Biologicals)
AF488 donkey anti goat (705-605-147, 1:200, Jackson Immuno Research)
AF647 donkey anti-rabbit (711-605-152 , 1:200, Jackson Immuno Research)
AF647 donkey anti-rat (712-605-153, 1:200, Jackson Immuno Research)
Ki-67 Monoclonal Antibody (SolA15) (eBioscience, 14-5698-80, 1:100)

Validation

All antibodies used in this study are commercially available. They are validated by the vendors for the specific assay and species used. The validation is available on the vendors website:
Anti-Mouse CD31: https://www.bdbiosciences.com/eu/applications/research/stem-cell-research/cancer-research/mouse/purified-rat-anti-mouse-cd31-mec-133/p/553370
Thrombospondin-4 Antibody: https://www.novusbio.com/products/thrombospondin-4-antibody-893655_mab7860
AF488 donkey anti goat: https://www.jacksonimmuno.com/catalog/products/712-605-153
AF647 donkey anti-rabbit: https://www.jacksonimmuno.com/catalog/products/711-605-152
AF647 donkey anti-rat: https://www.jacksonimmuno.com/catalog/products/712-605-153
Ki-67 Monoclonal Antibody: https://www.thermofisher.com/antibody/product/Ki-67-Antibody-clone-SolA15-Monoclonal/14-5698-82

Eukaryotic cell lines

Policy information about cell lines

Cell line source(s)

Gli1CreER;tdTomato mice were sacrificed two weeks after tamoxifen treatment.
Cardiac tdTomato+ cells were sorted by FACS and immortalized 14 days later with SVLargeT.
HEK293T cell-line, ATCC, #CRL3216, Lot:70008735, aliquots from passage 2 were used for the experiments.

Authentication

The gli1 cardiac fibroblast cell line was newly generated for this study and tdTomato expression measured by FACS confirmed its identity.

Mycoplasma contamination

All cell lines were tested negative for mycoplasma.

Commonly misidentified lines
(See ICLAC register)

No commonly misidentified cell lines were used.

Animals and other organisms

Policy information about studies involving animals; ARRIVE guidelines recommended for reporting animal research

Laboratory animals

PdgfrβCreERt2 (i.e.B6-Cg-Gt(Pdgfrβ-Cre/ERT2)6096Rha/J, JAX Stock #029684; n=9, 2 male, 7 female),
NG2CreER (B6.Cg-Tg(Cspg4-Cre/Esr1*)BAkik/J, JAX Stock #008538; n=6, 6 female),
Gli1CreER (Gli1tm3(Cre/ERT2)Alj/J, JAX Stock #007913; n=14, 7 male, 7 female),
Myh11CreER (B6.FVB-Tg(Myh11-Cre/ERT2)1Soff/J, JAX Stock #019079; n=6, 6 male)
were purchased from Jackson Laboratories (Bar Harbor, ME, USA).
C57Bl6/129SV-Collagen1alpha1-GFP-CreERT2 (Col1a1CreER, n=10, 4 male, 6 female)
was a kind gift of Ivica Grgic (Marburg).
Cdh5(PAC)-CreERT2 (Cdh5CreER ,Taconic no. 13073; n=12, 4 male, 8 female)
was a kind gift of Rui Benedito (Madrid). All Cre driver lines were crossbred with Rosa26tdTomato
(i.e. B6.Cg-Gt(ROSA)26Sortm9(CAG-tdTomato)Hze/J, JAX Stock #007909) also from
Jackson Laboratories, to generate the according tamoxifen inducible fate tracing genotype. All mice were included in the experiment at 8 weeks of age. Mice were housed with two to five animals per cage at a 12h light-dark cycle at sustained temperature (20°C ± 0.5 °C) and humidity (approximately 50% ± 10%) with ad libitum access to food and water.

Wild animals

This study did not involve any wild animals.

Field-collected samples

This study did not involve any samples collected from the field.

Ethics oversight	LANUV (Landesamt für Natur, Umwelt und Verbraucherschutz) North Rhine-Westphalia (Germany) approved the study protocol with reference No. 81-02.04.2018.A020.

Note that full information on the approval of the study protocol must also be provided in the manuscript.

Human research participants

Policy information about studies involving human research participants

Population characteristics	The human cardiac specimen was obtained from a 50-year-old male patient who underwent left ventricular assist device surgery due to ischemic cardiomyopathy.
Recruitment	Patient sample was selected based on the criteria relevant to our validation expermiment, therefore we think there was no selection bias.
Ethics oversight	The local ethics committee of the University Hospital RWTH Aachen approved all human tissue protocols (EK 151/09).

Note that full information on the approval of the study protocol must also be provided in the manuscript.

Flow Cytometry

Plots

Confirm that:

- [x] The axis labels state the marker and fluorochrome used (e.g. CD4-FITC).
- [x] The axis scales are clearly visible. Include numbers along axes only for bottom left plot of group (a 'group' is an analysis of identical markers).
- [x] All plots are contour plots with outliers or pseudocolor plots.
- [x] A numerical value for number of cells or percentage (with statistics) is provided.

Methodology

Sample preparation	FACS protocol and flow cytometry and detailed sample preparation are described in the Methods.
Instrument	SH800 Sorter (SONY) at University Hospital Aachen
Software	Sony FACS software (Version 2.1.6)
Cell population abundance	Post-sort purity was confirmed over 95%.
Gating strategy	Please see Methods and Supplementary Figure 12. Gating for all experiments: Cells (FSC-A vs. BSC-A), Singlets (BSC-A vs. BSC-W and FSC-A vs. FSC-W), Viable and tdTomato+ (DAPI vs. tdTomato), as fate traced cells were sorted. Gates and boundaries were defined by comparison to FMO and unstained samples.

- [x] Tick this box to confirm that a figure exemplifying the gating strategy is provided in the Supplementary Information.
